# Results of Preimplantation Genetic Testing for Aneuploidy (PGT-A) in a Cohort of 319 Embryos: Experience in a Fertility Clinic in Colombia

**DOI:** 10.5935/1518-0557.20210085

**Published:** 2022

**Authors:** Germán David Ospina Idárraga, Iván Darío Montes Suárez, Katherine Gisell Hernández Osorio, Diana Milena Diaz Corredor, Johanna Carolina Páez Redondo, Ricardo Garcia Yepes

**Affiliations:** 1 Instituto de Fertilidad Humana - Inser Bogotá, Colombia; 2 Clínica del Country - Bogotá, Colombia; 3 Master in Epidemiology Universidad del Rosario, Bogotá, Colombia; 4 Universidad del Rosario, Bogotá, Colombia

**Keywords:** aneuploidy, preimplantation genetic testing, biopsy, embryo

## Abstract

**Objective:**

To analyze the results of the preimplantation genetic testing for aneuploidy at the Instituto de Fertilidad Humana - Inser Bogotá, Colombia, from 2016 to 2020.

**Methods:**

This study is an observational, retrospective, and correlative analysis of biopsies from 319 embryos (from 54 patients) submitted to preimplantation genetic testing for aneuploidy by different molecular techniques.

**Results:**

Of the 54 patients included in the study, 42 provided their own oocytes, and 12 used donated oocytes. The main indication to perform the preimplantation genetic testing was advanced maternal age. We obtained 319 embryos: Ninety-one (28.5%) euploid, 197 (61.8%) aneuploid and 31 (9.7%) with no detectable DNA. The highest rate of aneuploid embryos was found in patients over 40 years (72.7%), and the euploidy rate in patients under 35 years was 37.1%. After the transfer of euploid embryos, the rates for implantation, ongoing pregnancy, live birth, and miscarriage were 40%, 50%, 40.6%, and 0%, respectively. Older maternal age correlated with higher numbers of aneuploid embryos and lower numbers of both euploid and 5-day embryos.

**Conclusions:**

There was a positive correlation between maternal age and aneuploidy rate. Complex chromosomal abnormalities were the most frequent aneuploidies, followed by mosaicism and double aneuploidies. The miscarriage rate after the transfer of euploid embryos was 0 %.

## INTRODUCTION

One of the leading causes of Assisted Reproduction Techniques (ART) failure is the transfer of aneuploid embryos (Wilton, 2002). Maternal age is a decisive factor in the generation of chromosomal alterations in the embryo; so that by the age of 35, the aneuploid embryo rate is significantly lower than in women over 40 years. After in vitro fertilization (IVF), the pregnancy rate is 31% in women under 35 years and just 10.2% in women over 40 years (Revelli *et al*., 2016). Embryo chromosomal abnormalities are more common in women of advanced reproductive age (ARA) (Franasiak *et al*., 2014; Harton *et al*., 2013; Milán *et al*., 2010; Schoolcraft *et al*., 2009); recurrent pregnancy loss (RPL) (Hodes-Wertz *et al.*, 2012; Vitez *et al.*, 2019); recurrent implantation failure (RIF) (Greco *et al*., 2014; Rubio *et al*., 2013a); severe male factor (SMF) (Practice Committees of the American Society for Reproductive Medicine & the Society for Assisted Reproductive Technology, 2018; Magli *et al*., 2009); and history of aneuploidies in the offspring (Harper *et al.*, 2010).

The morphological classification of embryos has been the most widely used method in IVF techniques to evaluate and stage embryo quality (Ebner *et al*., 2003). This method is limited to assessing morphological characteristics that are not necessarily related to euploidy (Ebner *et al*., 2003). The evolution of ART and the advances of molecular biology have enabled the development of new techniques to analyze the number of chromosomes and establish the embryo ploidy, optimizing the transfer of chromosomally healthy embryos (Ebner *et al*., 2003; Harper *et al*., 2010). Several molecular techniques have been used in recent years to determine the embryo's genetic quality, including Fluorescent In Situ Hybridization (FISH) (Álvarez Sedó, 2018; Wilton, 2002), Array Comparative Genomic Hybridization (aCGH) (Ata *et al*., 2012; Rubio *et al*., 2013b; Rodrigo *et al*., 2014), quantitative real-time PCR (qPCR), Single Nucleotide Polymorphisms (SNP), and Next Generation Sequencing (NGS) (Fiorentino *et al*., 2014; Kung *et al*., 2015; Palmerola *et al*., 2019). The aCGH analyzes the entire genome with a high resolution; it is a powerful, agile, and highly specific tool suitable to diagnose chromosome gains; however, it cannot detect point mutations or balanced rearrangements (Shinawi & Cheung, 2008). The NGS enables the simultaneous screening of millions of DNA or RNA sequences and performs several analyses simultaneously in the same embryo (monogenic disorders, structural alterations, and aneuploidies). This technique has better accuracy, high performance and sensibility to detect genetic variants of low frequency (Zhong *et al*., 2021).

These molecular techniques require an embryo biopsy obtained at different developmental stages (Harper *et al*., 2010). According to results from randomized controlled trials, the 2012 European Preimplantation Genetic Diagnosis (PGD) Consortium advised against taking biopsies from blastomeres and against the use of the FISH technique; likewise, it recommended the trophectoderm biopsy and the chromosomal material analysis by NGS (Harper *et al*., 2010).

At the PGD European Consortium, preimplantation genetic testing for aneuploidy (PGT-A) was reported for 6,095 cycles of IVF. The test was indicated in cases of ARA (40%), RIF (12%), ARA and RIF (12%), ARA and RPL (10%), and SMF (10%). Other indications were previous abnormal pregnancies, impaired paternal or maternal karyotype including mosaicisms; a small number of couples did not have a medical indication (Harper *et al.*, 2012).

Since 1990, pregnancies resulting from the transfer of IVF-embryos previously tested by molecular techniques have been documented and accounted for approximately 11,000 live births by 2010 (Simpson, 2010). However, there are controversial reports; some of them support (Chen *et al.*, 2015; Coates *et al*., 2017; Rubio *et al*., 2013a; Scott *et al*., 2013; Wilton, 2002; Yang *et al*., 2012) but others discourage (Chang *et al*., 2016; Kang *et al*., 2016; Mastenbroek *et al*., 2011) the use of PGT-A as a strategy to increase both implantation and live birth rates. This study aimed to analyze the PGT-A results reported at the Instituto de Fertilidad Humana - Inser Bogotá, Colombia.

## MATERIALS AND METHODS

This study was an observational, retrospective and correlative analysis of the PGT-A results of embryo biopsies collected between February 2016 and March 2020 at the Instituto de Fertilidad Humana - Inser Bogotá, Colombia.

The study was approved by the Medical and Research Ethics Committee of the Instituto de Fertilidad Humana - Inser Bogotá, ensuring the study's compliance vis-à-vis the ethical standards and those defined in the 1975 Helsinki declaration, revised in 2013. All patients filled out and signed an institutional and reference laboratory informed consent form, accepting the procedures, risks and possible complications. Fifty-four patients gave their consent for 319 biopsies to be taken.

### Ovarian hyperstimulation, fertilization, and embryo culture

Controlled ovarian hyperstimulation (COH) was performed under a flexible Gonadotropin-releasing hormone antagonist (GnRH) protocol. Gonadotropin doses were adjusted according to age, body mass index, and ovarian reserve testing. The oocytes retrieved through follicular aspiration were cultured in Life Global Total medium/HEPES (Life Global^®^) or GMops Plus (Vitrolife^®^) for two hours until decumulation using ICSIcumulase (Origio^®^) or Hyase (Vitrolife^®^). Subsequently, the ICSI was conducted, and the injected oocytes were cultured in NUNC™ plates (CN 150255 Nunc^®^) in Life Global Total or GTL plus (Vitrolife^®^) culture medium under a mineral oil layer (LiteOil; Life Global^®^ or OVOIL; Vitrolife^®^), and incubated at a pH of 7.3, 5% oxygen and 8,9% CO2 in Tri-Gas incubators (K-Systems^®^). Until August 2018, Life Global^®^ media were used; but since then, and following changes in the laboratory's internal protocols, Vitrolife^®^ media is being used.

After ICSI, fertilization was assessed between 16 and 18 hours; embryo culture continued for 72, 120 or 144 hours according to the embryo development. The 72-hour embryos were classified according to their blastomere number and fragmentation percentage (ASEBIR, 2015), while 120- and 144-hour embryos were graded according to the Gardner and Schoolcraft's system (Gardner *et al*., 1998).

### Embryo biopsy

The 72-hour embryos were selected only if they had between 7 and 10 blastomeres and a fragmentation lower than 15%. These embryos were cultured in NUNC(tm) plates (CN 150265; Nunc) using Life Global PGD Biopsy Medium (Life Global^®^) and under a mineral oil layer (LiteOil or OVOIL). The embryo was held using a holding pipette (Origio^®^ or RI^®^) on an inverted microscope equipped with a micromanipulation system. A mononuclear blastomere was chosen, and its zona pellucida was laser-drilled (Lykos^®^; Hamilton Thorne, USA) before aspiration, using a biopsy pipette (Origio^®^). The retrieved cell was cultured into Life Global Total/HEPES medium or in GMops Plus.

Since July 2017, modifications to InSer's internal protocols established that trophectoderm biopsies be held at 120 and 144 hours for all the embryos to be analyzed by PGT-A. For this reason, the embryos were cultured on NUNC™ plates (CN 150265; Nunc) with Life Global Total medium/HEPES or GMops Plus under a mineral oil layer (LiteOil or OVOIL;). Before the biopsy (12 to 24 hours), the zona pellucida was laser-pierced (Lykos-^®^; Hamilton Thorne). Then, using an inverted microscope equipped with a micromanipulation system, the blastocysts were fixed by a holding pipette, and 6 to 10 cells were aspirated with a microinjector and the biopsy pipette. The cells were then severed by 3 to 4 diode laser shots at 500 pulses per microsecond or flicking. The cells obtained in the biopsy were released into Life Global Total/HEPES medium or GMops Plus.

For tubing (cell isolation), the aspirated trophectoderm cells were washed with sterile phosphate-buffered saline solution and transferred to sterile microtubes containing an isolation medium for molecular testing. A total of 319 embryo biopsies were split to be sent to different laboratories for chromosomal analysis as follows: 136 biopsies to Igenomix Laboratories in the United States of America (76 for NGS and 60 for aCGH), 142 to Sistemas Genómicos in Spain (for NGS), and 28 and 13 respectively to Colgenes and Genetix in Colombia to be analyzed by NGS.

The laboratories used to classify these chromosomal abnormalities as numerical and segmental according to internal criteria. It is important to note that the chromosomal abnormalities mostly reported are monosomy, trisomy, deletion or duplication, and mosaicism. These alterations can be combined, and depending on the number, they are called double, complex, and chaotic. For this study, the combined abnormalities were classified as double (two numerical or segmental alterations), complex (three to four abnormalities), and chaotic (five or more alterations).

### Vitrification and thawing

Vitrification was performed using the Cryotech^®^ kit following the manufacturer›s protocols. All the embryos were vitrified immediately after performing the biopsy. The Euploid embryos were thawed using the Cryotech^®^ kit and cultured in Life Global Total or GTL plus medium between 4 and 6 hours before their transfer. On the other hand, the embryos that were biopsied at 72 hours of development were further cultured until the blastocyst stage for transfer purposes.

### Embryo transfer and pregnancy confirmation

Before embryo transfer, the recipient women were subjected to an endometrial preparation protocol with GnRH agonists and oral estrogens (progressive doses until reaching 6 to 8 mg/day of Progynova, Bayer^®^). When the expected endometrial thickness was achieved, treatment with progesterone (600 to 800 mg/day) was initiated. On the 5^th^ day of progesterone administration, one or two embryos were transferred through the cervical canal into the uterine cavity using a Cook^®^ transfer catheter under transabdominal ultrasound guidance.

The pregnancy test was performed 14 days after embryo transfer by quantitative determination of Human Chorionic Gonadotropin beta subunit (b-HCG) in the serum. Ultrasound monitoring was carried out at six weeks of pregnancy, and prenatal care was continued in the positive cases.

### Data Analysis

This study analyzed data from women who underwent PGT-A at the Instituto de Fertilidad Humana- InSer, Bogotá between February 2016 and March 2020. The analyses were performed in a total of 319 embryos. Their medical records were reviewed, and the following variables were analyzed: patient age, indication for PGT-A, oocyte origin, total gonadotropin dose administered, the total number of mature and immature oocytes collected, total number of 72-, 120-, and 144- hour embryos, number of biopsied embryos, PGT-A results, molecular technique used for PGT-A, number of transfers, number of embryos transferred, number of patients who received embryos, pregnancy test result, number of gestational sacs visualized, and number of live births.

Statistical analyses were run in the SPSS v22.0 software. We ran a descriptive analysis using absolute and relative frequencies for qualitative variables, and mean and standard deviation (SD), or median and interquartile range (IQR) for qualitative variables, depending on data distribution. The normality of variable distribution was analyzed by the Shapiro-Wilk and Kolmogorov- Smirnov tests. The correlations between variables were determined by the Pearson or Spearman correlation coefficients, with a statistically significant *p-*value < 0.05; according to the coefficient calculated, the correlation was interpreted as very low (0.00-0.19); low (0.20-0.39), moderate (0.40-0.59), good (0.60-0.79), and very good (0.80-1.00).

Additionally, the rates for implantation, ongoing pregnancy per transferred patients, live birth per transferred patients, and miscarriage were calculated according to the following formulas: $\mathrm{Implantation}\;\mathrm{rate}=\left(\mathrm{number}\;\mathrm{of}\;\mathrm{gestational}\;\mathrm{sacs}\;\mathrm{observed}/\mathrm{number}\;\mathrm{of}\;\mathrm{transferred}\;\mathrm{embryos}\right)\;X\;100.$ $\mathrm{Ongoing}\;\mathrm{pregnancy}\;\mathrm{rate}\;\mathrm{per}\;\mathrm{transferred}\;\mathrm{patients}=\left(\mathrm{number}\;\mathrm{of}\;\mathrm{patients}\;\mathrm{with}\;\mathrm{at}\;\mathrm{least}\;\mathrm{one}\;\mathrm{gestational}\;\mathrm{sac}\;\mathrm{after}\;\mathrm{week}\;5\;\mathrm{with}\;\mathrm{fetal}\;\mathrm{heartbeat}/\;\mathrm{number}\;\mathrm{of}\;\mathrm{transferred}\;\mathrm{patients}\right)\;X\;100.$ $\mathrm{Live}\;\mathrm{birth}\;\mathrm{rate}\;\mathrm{per}\;\mathrm{transferred}\;\mathrm{patients}=\left(\mathrm{number}\;\mathrm{of}\;\mathrm{births}\;\mathrm{resulting}\;\mathrm{in}\;\mathrm{at}\;\mathrm{least}\;\mathrm{one}\;\mathrm{live}\;\mathrm{birth}\;/\mathrm{number}\;of\;\mathrm{transferred}\;\mathrm{patients}\right)\;X\;100.$ $\mathrm{Miscarriage}\;\mathrm{rate}=\left(\mathrm{number}\;\mathrm{of}\;\mathrm{patients}\;\mathrm{with}\;\mathrm{pregnancy}\;\mathrm{loss}\;\mathrm{by}\;\mathrm{week}\;12/\left(\mathrm{number}\;\mathrm{of}\;\mathrm{patients}\;\mathrm{presenting}\;\mathrm{at}\;\mathrm{least}\;\mathrm{one}\;\mathrm{gestational}\;\mathrm{sac}\;\mathrm{after}\;\mathrm{week}\;5\right)\;X\;100.\right.$

## RESULTS

The study included 54 women: 42 patients (median age 40 years; IQR = 37-42) provided their own oocytes, and 12 (median age 24.5 years; IQR = 22.2-26.7) used donated oocytes. All oocytes provided accounted for a total of 319 embryos (Table 1).

**Table 1.** Distribution of patients by age.

Age range (years)	Number of patients (%)
< 35	15 (27.8)*
35 - 37	10 (18.5)
38 - 40	11 (20.4)
> 40	18 (33.3)
Total	54

* Including 12 women who were given embryos produced from donated oocytes

The analysis found that the main indications for PGT-A were ARA, the combination of 2 or 3 indications, and screening, followed by RIF and RPL as unique indications (Table 2). In the group of 42 patients who provided their own oocytes (some required up to 3 COH cycles), the number of oocytes collected reached a median of 14.5 (IQR = 9-24.2): 11 (IQR = 7-17) oocytes at metaphase II and 3.5 (IQR = 1.75-6.25) immature oocytes.

**Table 2.** Distribution of patients by PGT-A indication.

Indication for PGT-A	Number of patients	Percentage (%)
ARA	21	38.9
Combination of 2 or 3 indications *	14	25.9
Screening	13	24.1
RIF	3	5.6
RPL	2	3.7
Family history of aneuploid pregnancies †	1	1.8
Total	54	100

* Combinations found: ARA/RPL: 4; ARA/RIF: 1; ARA/personal or family history: 1; RIF/RPL: 1; screening/personal or family history: 3; RIF/personal or family history: 1; RPL/personal or family history: 1; ARA/RIF/RPL: 1; ARA/RPL/ personal or family history:1.

† Family history of aneuploid pregnancies: patient with a second degree relative with Down syndrome.

A total of 319 embryos were produced *in vitro* with a median of 5 (IQR = 2.7-7.2) embryos per patient. Of these embryos, 91 were euploid, 197 aneuploid, and 31 could not be classified because DNA was not detected in the samples (Table 3).

**Table 3.** PGT-A results.

PGT-A result	Number of cases	Frequency (%)
Euploid Embryos	91	28.5
Abnormal Embryos	197	61.8
Undetected DNA	31	9.7
Total	319	100

The chromosomal alterations identified were mainly complex anomalies followed by mosaicism and double aneuploidies (Table 4). Monosomies were mainly located on chromosomes 15, 16, and 18, and trisomy in chromosomes 15, 21 and 22.

**Table 4.** Aneuploidies reported in abnormal embryos.

Aneuploidy	Number of cases	Frequency (%)
Monosomies	30	15.2
Trisomy	24	12.2
Double aneuploidy	30	15.2
Deletion or Duplication	19	9.6
Mosaicism	36	18.3
Complex aneuploidy	52	26.4
Chaotic aneuploidy	6	3.1
Total	197	100

Most biopsied embryos came from women under 35 years of age and exhibited the highest euploidy rate. In contrast, the highest rate of aneuploid embryos was found in patients over 40 years of age (Table 5).

**Table 5.** Distribution of patients by age group and PGT-A results of embryos.

Women age	Embryos screened by PGT-A						
	Total	Euploid		Aneuploid		Undetectable DNA	
(years)	(n)	(n)	(%)	(n)	(%)	(n)	(%)
< 35	105	39	37.1	58	55.2	8	7.7
35 - 37	88	27	30.7	50	56.8	11	12.5
38 - 40	60	14	23.3	41	68.4	5	8.3
> 40	66	11	16.7	48	72.7	7	10.6

Biopsies taken from 60 embryos (18.8%) were analyzed by aCGH; this group consisted of embryos cultured for different hours as follows: 72-h (n=37), 120-h (n=21), and 144-h (n=2). The abnormalities most frequently observed were complex aneuploidies followed by segmental aneuploidies and monosomies (Tables 6 and 7). Seven of these 60 embryos were transferred to 6 patients, and two positive pregnancy tests were detected afterwards.

**Table 6.** PGT-A results according to the molecular technique.

PGT-A result	Molecular technique			
	aCGH		NGS	
	(n)	%	(n)	%
Normal embryos	12	20	79	30.5
Abnormal embryos	42	70	155	59.8
Undetected DNA	6	10	25	9.7
Total	60	100	259	100

**Table 7.** Aneuploidies found in embryos according to the molecular technique.

PGT-A result	Molecular technique			
	aCGH		NGS	
	(n)	%	(n)	%
Monosomies	8	19.0	22	14.2
Trisomy	4	9.5	20	12.9
Double aneuploidy	6	14.3	24	15.5
Deletion or duplication	11	26.2	8	5.2
Mosaicism	0	0	36	23.2
Complex Aneuploidy	12	28.6	40	25.8
Chaotic Aneuploidy	1	2.4	5	3.2
**Total**	42	100	155	100

On the other hand, biopsies taken from 259 (81.2%) embryos were analyzed by NGS; this group also contained embryos cultured for different hours: 72-h (n=9), 120-h (n=194) and 144-h (n=56). The most common anomalies found were complex aneuploidies followed by mosaicism and double alterations; a lower number of segmental alterations were detected. The percentage of embryos with undetectable DNA was similar in both molecular techniques (Tables 6 and 7). The transfer of 38 of these 259 embryos to 26 patients resulted in 15 positive pregnancy tests.

Forty-five of the 91 euploid embryos obtained were used in 33 transfers to 32 patients with a resulting median for embryo transfer of 1 (IQR = 1-2); 19 (59.3%) patients underwent single embryo transfer (SET) and 13 (40.6 %) received two embryos. Afterwards, there were 17 positive pregnancy tests. Forty-six euploid embryos have not yet been transferred and are kept vitrified.

Rates for implantation, clinical pregnancy, live birth, and miscarriage were calculated in this study (Table 8). Besides, the implantation rates according to the molecular technique were 14.2 % and 44.7 % for aCGH and NGS, respectively.

**Table 8.** Implantation, clinical pregnancy, live birth and miscarriage rates.

Estimated rate	Overall Result
Implantation rate	40.0%
Ongoing pregnancy rate	50.0%
Live birth rate	40.6%
Miscarriage rate	0.0%

Correlation analysis between age and other variables showed that the older the patient, the greater the number of aneuploid embryos (low positive correlation), the lower the numbers of 120-hou embryos (good negative correlation), the lower the euploid embryos (moderate negative correlation) (Table 9).

**Table 9.** Correlation Analysis.

Dependent variable	Independent variables	Rho Spearman	*p*-value
Age	Total gonadotropin dose	0.227	0.15
	Total number of oocytes retrieved	-0.261	0.09
	Total number of oocytes, metaphase II	-0.308	**0.047**
	Total number of 3-day embryo	0.147	**0.348**
	Total number of 5-day embryo	-0.677	**0.000**
	Total number of 6-day embryo	0.175	0.206
	Total vitrification	-0.439	**0.001**
	Number of embryos/patient	-0.535	**0.000**
	% normal embryos	-0.440	**0.001**
	% abnormal embryos	0.385	**0.004**

## DISCUSSION

Throughout the history of reproductive medicine, many ART-optimizing techniques have been introduced. Among them, IVF, ICSI, vitrification, and PGT-A approaches are worth mentioning (Álvarez Sedó, 2018). Since its introduction, the PGT-A has emerged as a tool to improve pregnancy rates compared to other approaches, where no embryo genetic testing was performed.

A well-known feature of the human species is the increased generation of aneuploid embryos as the maternal age advances; this characteristic gives rise to lower implantation and pregnancy rates, increased miscarriages and chromosomal diseases (Ata *et al*., 2012; Harton *et al*., 2013). The main objective of PGT-A is the selection of euploid embryos, intended to increase the live birth rate by promoting the SET and reducing the time to achieve a successful pregnancy (Álvarez Sedó, 2018). However, the initial results that seemed to be promising to have gradually lost their applicability due to controversial reports in different recent publications.

The present study showed increased aneuploidy rates in embryos from older women: 72.7 % of aneuploid embryos were identified in patients over 40 years of age. There is similar data in the literature, e.g., the study by Harton *et al.* (2013) reported that in women over 40 years, the aneuploidy rates in embryos biopsied on days 3 and 5 were 85.8% and 76.3%, respectively. Another study of 15,169 embryos found aneuploidy rates of 58% among patients older than 40 years and a steadily increasing rate up to approximately 85% in 43-year-old patients (Franasiak *et al*. 2014).

The genetic abnormalities mainly identified in aneuploid embryos were double, complex, and numerical in patients over 38 years of age; and segmental in those under 35 years. As shown by some studies, numerical aneuploidies increase with age while segmental or single chromosome alterations decrease (Franasiak *et al*., 2014; Sánchez-Usabiaga *et al*., 2017). Moreover, complex abnormalities progressively increase with age, as reported in the study by Rodrigo *et al.* (2014), where two groups of embryos were analyzed; the first group included 3,146 embryos, from women under 40 years, in which 14.2% of chaotic aneuploidies and 20.8% of double and complex aneuploidies were detected; in the second group, 3,972 embryos from women over 40 years of age exhibited 15.6% of chaotic aneuploidies and 43.1% of double and complex aneuploidies.

In the current study, the main indication for PGT-A was the advanced maternal age (46.4%). In this group of patients, it is essential to identify and select healthy embryos to be transferred to increase implantation and decrease the miscarriage rates. Older women not only have a higher risk of producing aneuploid embryos, but also a lower number of embryos owing to their low ovarian reserve and oocyte quality; all these factors affect embryo development, making it difficult to obtain blastocysts suitable for PGT-A (Harton *et al*., 2013). As an alternative, some patients choose to undergo treatment using donated oocytes; nevertheless, some studies advise against the use of PGT-A in these cases due to the lack of significant differences in pregnancy and miscarriage rates when comparing embryos (produced from donated oocytes) screened or not by PGT-A (Haddad *et al*., 2015).

In patients with RIF, PGT-A increases the chance of pregnancy by the transfer of euploid embryos (Forman *et al*., 2013; Greco *et al*., 2014; Rubio *et al*., 2013a). However, it is important to note that other factors (endometrial receptivity, inadequate expression of endometrial adhesive molecules, autoimmune diseases and uterine physiological alterations) can affect the embryo's ability to develop, hatch and implant and, consequently, hinder the growth of even euploid embryos (Achache & Revel, 2006). This issue was supported by a study of patients with RIF by Blockeel *et al*. (2008) who did not find significant differences in pregnancy rates after transferring them with embryos tested or not by PGT-A. In the present study, in patients with RIF, the aneuploidy rate was 62.5%; and the pregnancy test was positive in 71.4% of cases after transfer of euploid embryos.

One of the main causes of RPL is the presence of aneuploid embryos; in this study, RPL was an indication for using PGT-A in 18.5% of patients. Several studies have demonstrated a decrease in the miscarriage rate when PGT-A is performed (Hodes-Wertz *et al.*, 2012; Simon *et al.*, 2018). Furthermore, in couples suffering from RPL with a history of genetic abnormalities, PGT (PGT-A, PGT-M, PGT-SR) is an option for aneuploidy screening, monogenic diseases and structural and numerical chromosomal abnormalities; it avoids the transfer of abnormal embryos and the consequent risk of miscarriage (ESHRE, 2017). In this study, most patients had an indication to request a genetic study and only 24.1% of them requested a PGT-A for screening purposes, which explains the high rate of aneuploidy here reported.

In the current study, the implantation rate was 40% after the transfer of euploid embryos; similar figures were published in cases not screened by PGT-A by the REDLARA (Red Latinoamericana de reproducción asistida) in 2017. It reported implantation rates of 37.8% after transfer of own-thawed embryos, and 43.7% for embryos produced from donated oocytes (Zegers-Hochschild *et al*., 2020a;b). Notwithstanding, other studies support the relevance of PGT-A in the improvement of embryo implantation; for instance, a study found implantation rates of 63.2% in patients who were given euploid embryos and of 51.2% in those who received two blastocysts without PGT-A (Forman *et al.*, 2013). Another study published implantation rates of 66.4% and 47.9% after the transfer of embryos with or without PGT-A screening, respectively (Scott *et al*., 2013).

In the present study, the implantation rate was higher when the PGT-A was performed by NGS than by aCGH. This difference may be attributed to the higher efficiency of NGS and the timing of the introduction of aCGH at InSer, which coincided with implementing the genetic testing analysis program; therefore, some factors such as skill and expertise could have affected the results.

According to the study by Homer (2019), the live birth rate in cases with PGT-A does not vary significantly since the embryo potential is not increased by the PGT-A itself; however, miscarriage rates may be reduced by performing the PGT-A For instance, the study by Yang *et al.* (2012) found clinical pregnancy rates of 70.9% in cases of embryos graded by morphology and screened for chromosomal anomalies by aCGH, and 45.8% in cases of embryos selected only by morphological criteria. In a similar study, clinical pregnancy rates were 52.1% and 34.9% when embryos had been screened respectively by PGT-A or morphology on day five (Rubio *et al*., 2013a). In the present study, the ongoing pregnancy rate in cases with PGT-A was 50%, a figure consistent with data from other studies reporting significant improvement in ongoing pregnancy rates when PGT-A was performed (Ata *et al*., 2012; Chen *et al.*, 2015; Coates *et al*., 2017; Harper *et al*., 2012; Hodes-Wertz *et al*., 2012; Munné *et al*., 2016).

The miscarriage rates reported by the REDLARA in 2017 when PGT-A had been performed were 12.8%, 13.9%, and 9.3% in women under 35 years, between 35 and 39, and over 39 years, respectively; in contrast, in cases without the PGT-A screening, the figures for the same age groups were 15.3%, 17.8%, and 23.7%, respectively (Zegers-Hochschild *et al*., 2020a;b). In the present study, the miscarriage rate upon PGT-A was 0%, but this figure could have been minimized due to the limited number of patients analyzed. Notwithstanding, the PGT-A could be a useful tool to reduce the time to pregnancy for patients with RPL. As the literature shows, several studies described that PGT-A considerably reduces the miscarriage rate (Practice Committees of the American Society for Reproductive Medicine & the Society for Assisted Reproductive Technology, 2018; Harton *et al.*, 2013; Homer, 2019). A significant reduction in the miscarriage rate from 26% to 10% in women younger than 35 years of age and from 39% to 13% in those over 35 years was reported in a study by Hodes-Wertz *et al*. (2012). Additionally, a multicenter study in patients over 35 years found miscarriage rates of 7% and 14% in cases with and without PGT-A, respectively (Verpoest *et al*., 2018).

The reliability and success of PGT-A depend not only on the proper functioning of a reproductive clinic but also on the expertise of the laboratory personnel and suitable sample processing and analysis. Among the study's limitations are the impossibility of controlling some variables due to the retrospective nature of the study; besides, PGT-A was not randomly indicated because, in some cases, it was chosen by the patients.

## CONCLUSIONS

PGT-A is a screening approach for embryos, being frequently used in the area of reproductive medicine. Over time, PGT-A has been indicated for some specific groups: patients over 40 years of age, recurrent pregnancy loss, recurrent implantation failure, and history of inherited genetic abnormalities. In this study, the aneuploidies most frequently found in embryos were complex chromosomal abnormalities followed by mosaicism and double aneuploidies. Besides, a positive correlation was found between maternal age and aneuploidy rate. It is noteworthy that the miscarriage rate found was 0% upon transfer of euploid embryos, previously identified by PGT-A.
